# The Sufferings of Christ

**Published:** 1888-02-25

**Authors:** 


### Words of Consolation.

[Communications for this column should be addressed "Chaplain," care of the Editor of The Hospital, who will gratefully welcome any suggestions or inquiries from matrons, nurses, and the staff generally.]

### LXVII.—The Sufferings of Christ.

*For Reading to the Sick.*

Sin separates the soul from God. Every sin is in itself an act of separation. Habitually sinful, careless lives constitute a desertion of God. It becomes clear in this way that Hell is but the outgrowth of this desertion. The lot of the ungodly in the world to come is represented broadly as "everlasting destruction *from the presence* of the Lord" (2 Thess. i. 9). "Broad is the way that leadeth to destruction" (Matt. vii. 13). The destruction consists in the desertion, not in the annihilation of the wicked, as some would teach us now. A careful examination of the passages which speak of the destruction of the wicked in fair comparison with others bearing upon future retribution, must convince any reasonable person that separation from God is the root idea conveyed. There will be desolation in outer darkness, because from him that hath not used the light while it was day shall be taken away even that which he had. Our Lord speaks of killing; but, after He has killed, the existence of the person so visited continues. "I say unto you, my friends, be not afraid of them that kill the body, and after that have no more that they can do (fear not dissolution). But I will forewarn you whom ye shall fear: Fear him which after he hath killed hath power to cast into hell; yea, I say unto you, Fear him" (fear desolation) (Luke xii. 4, 5). Where, then, lay the very taste of death for our Lord, in so far as it was possible for His innocent soul to experience such a curse? How was He "made sin for us"? (2 Cor. v. 21.) Surely in the utter desolation, in the Great Darkness, in the veiling of the Father's presence, in the agony of feeling as if He were deserted by God. Thus did He allow Himself of His own free will, for our sakes, and for our salvation, to endure a taste of the extreme consequence of sin. We must be careful to note that this sense of desertion could only have been in feeling, not in fact. For God to have been separated from God, or, indeed, from the manhood taken into God, was not possible. He who tasted death, and plunged as very man into the abyss of spiritual darkness, is Light of Light, Very God of Very God. Yet, if we would fully accept the mystery of the Atonement, we shall believe nothing less than that He became familiar with essence of the second death. "My God! my God! Why hast Thou forsaken me?" Why? Does He not seem to answer all who would seek an answer to that awful question, Why hast Thou? "Surely that there may issue an appeal to the world from this depth of My woe such as none but the utterly hardened shall be able to resist. O Father, if they have ever known this tale of My sorrow, and yet refused so great salvation; if they have beheld Thee so far from My health and from the words of My complaint; if they have heard my exceeding bitter cry in the daytime—*the daytime of their life*—and not been moved by it; and Thou continuest holy, Thou who are from everlasting: surely there will be a night season for them when they will take no rest."

